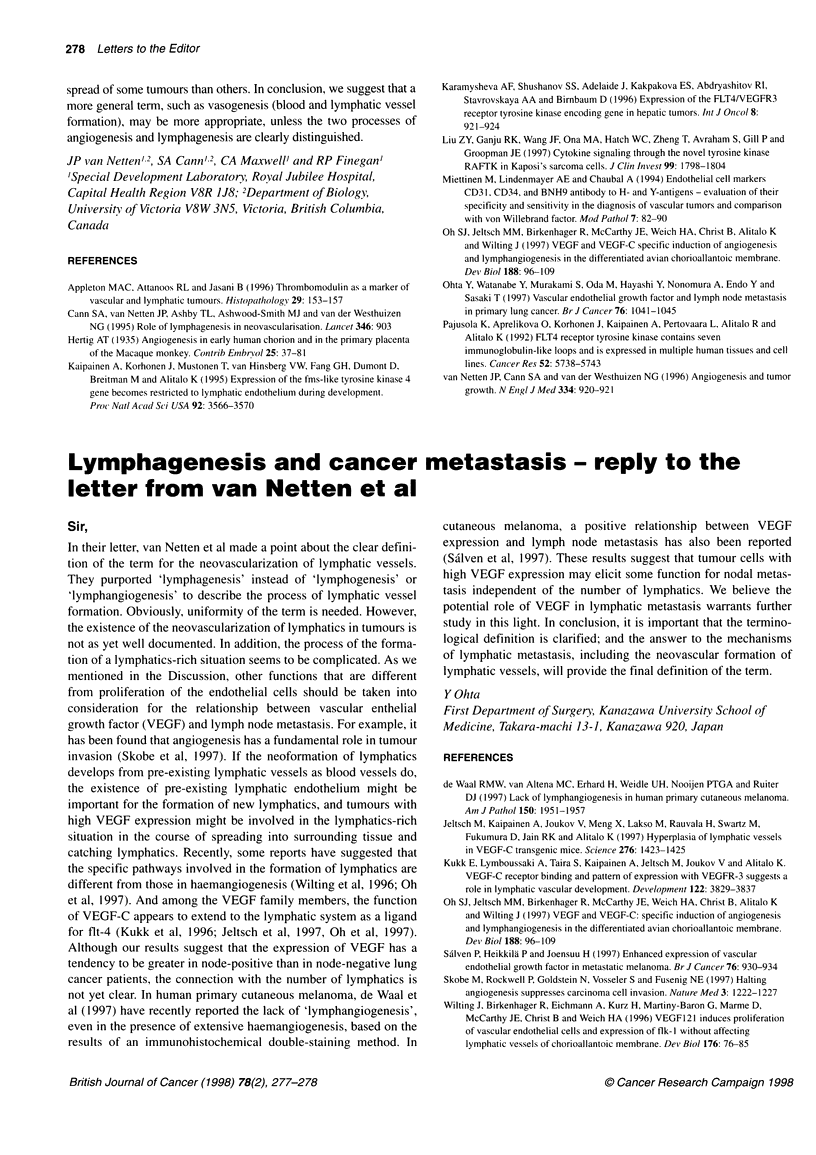# Lymphagenesis and cancer metastasis - reply to the letter from van Netten et al

**Published:** 1998-07

**Authors:** Y Ohta


### Lymphagenesis and cancer metastasis – reply to the letter from van Netten et al

**Sir,**

In their letter, van Netten et al made a point about the clear definition of the term for the neovascularization of lymphatic vessels. They purported 'lymphagenesis' instead of 'lymphogenesis' or 'lymphangiogenesis' to describe the process of lymphatic vessel formation. Obviously, uniformity of the term is needed. However, the existence of the neovascularization of lymphatics in tumours is not as yet well documented. In addition, the process of the formation of a lymphatics-rich situation seems to be complicated. As we mentioned in the Discussion, other functions that are different from proliferation of the endothelial cells should be taken into consideration for the relationship between vascular enthelial growth factor (VEGF) and lymph node metastasis. For example, it has been found that angiogenesis has a fundamental role in tumour invasion (Skobe et al, 1997). If the neoformation of lymphatics develops from pre-existing lymphatic vessels as blood vessels do, the existence of pre-existing lymphatic endothelium might be important for the formation of new lymphatics, and tumours with high VEGF expression might be involved in the lymphatics-rich situation in the course of spreading into surrounding tissue and catching lymphatics. Recently, some reports have suggested that the specific pathways involved in the formation of lymphatics are different from those in haemangiogenesis (Wilting et al, 1996; Oh et al, 1997). And among the VEGF family members, the function of VEGF-C appears to extend to the lymphatic system as a ligand for flt-4 (Kukk et al, 1996; Jeltsch et al, 1997, Oh et al, 1997). Although our results suggest that the expression of VEGF has a tendency to be greater in node-positive than in node-negative lung cancer patients, the connection with the number of lymphatics is not yet clear. In human primary cutaneous melanoma, de Waal et al (1997) have recently reported the lack of 'lymphangiogenesis', even in the presence of extensive haemangiogenesis, based on the results of an immunohistochemical double-staining method. In cutaneous melanoma, a positive relationship between VEGF expression and lymph node metastasis has also been reported (Sálven et al, 1997). These results suggest that tumour cells with high VEGF expression may elicit some function for nodal metastasis independent of the number of lymphatics. We believe the potential role of VEGF in lymphatic metastasis warrants further study in this light. In conclusion, it is important that the terminological definition is clarified; and the answer to the mechanisms of lymphatic metastasis, including the neovascular formation of lymphatic vessels, will provide the final definition of the term.

*Y Ohta*
*First Department of Surgery, Kanazawa University School of Medicine, Takara-machi 13-1, Kanazawa 920, Japan*

### REFERENCES

de Waal RMW, van Altena MC, Erhard H, Weidle UH, Nooijen PTGA and Ruiter DJ (1997) Lack of lymphangiogenesis in human primary cutaneous melanoma. *Am J Pathol* **150**: 1951–1957
Jeltsch M, Kaipainen A, Joukov V, Meng X, Lakso M, Rauvala H, Swartz M, Fukumura D, Jain RK and Alitalo K (1997) Hyperplasia of lymphatic vessels in VEGF-C transgenic mice. *Science* **276**: 1423–1425
Kukk E, Lymboussaki A, Taira S, Kaipainen A, Jeltsch M, Joukov V and Alitalo K. VEGF-C receptor binding and pattern of expression with VEGFR-3 suggests a role in lymphatic vascular development. *Development* **122**: 3829–3837
Oh SJ, Jeltsch MM, Birkenhager R, McCarthy JE, Weich HA, Christ B, Alitalo K and Wilting J (1997) VEGF and VEGF-C: specific induction of angiogenesis and lymphangiogenesis in the differentiated avian chorioallantoic membrane. *Dev Biol* **188**: 96–109
Sálven P, Heikkilä P and Joensuu H (1997) Enhanced expression of vascular endothelial growth factor in metastatic melanoma. *Br J Cancer* **76**: 930–934
Skobe M, Rockwell P, Goldstein N, Vosseler S and Fusenig NE (1997) Halting angiogenesis suppresses carcinoma cell invasion. *Nature Med* **3**: 1222–1227
Wilting J, Birkenhager R, Eichmann A, Kurz H, Martiny-Baron G, Marme D, McCarthy JE, Christ B and Weich HA (1996) VEGF121 induces proliferation of vascular endothelial cells and expression of flk-1 without affecting lymphatic vessels of chorioallantoic membrane. *Dev Biol* **176**: 76–85

© Cancer Research Campaign 1998